# The history of the JACMP debate series

**DOI:** 10.1002/acm2.14446

**Published:** 2024-06-23

**Authors:** Yi Rong, Jay Burmeister

**Affiliations:** ^1^ Mayo Clinic Arizona Radiation Oncology Mayo Blvd Phoenix Arizona USA; ^2^ Wayne State University School of Medicine/Barbara Ann Karmanos Cancer Institute Detroit Michigan USA

Physics is the field of science that explores the structure of matter and the interactions between the basic components of the universe. In contrast, medicine is a discipline rooted in practice, experience, and clinical findings, characterized by inherent variation and personalization. Combining physics and medicine forms the foundation of our field, Medical Physics. In a sense, Medical Physics is a blend of art and science. Becoming a good medical physicist involves mastering the art of balancing conflicting, even contradictory, objectives.

The existence of the medical physics discipline in healthcare is quite unique. The recruitment of scientists trained in various physical sciences to develop novel medical techniques helped to create the medical specialties of diagnostic imaging, nuclear medicine, and radiation oncology as we know them today. Ultimately, this novelty grew to become a mature profession which is now comprised of a dedicated group of scientists that continues to propel these fields forward at a rapid pace. As the field continues to evolve, new scientific challenges emerge with which we must grapple.

By questioning and probing ideas, we drive innovation and challenge the status quo. This process allows us to explore alternate angles and think about subjects in multiple ways, facilitating the progress of science. The practice of medicine is safer and more effective today as a result of such vetting through scientific debate, the ultimate goal being the improvement in the lives of our patients.

In 2015, JACMP launched an editorial series called “Parallel Opposed (PO)” to highlight these controversial topics, presenting arguments from various perspectives and inspiring readers to contemplate the balance between art and science in our field. The first PO article debated the use of log‐file QA versus measurement‐based QA for IMRT.^1^ To this date, JACMP has published almost forty PO debate editorials, covering a wide range of topics on clinical practice, education, profession, and beyond. In 2019, a novel team debate format was initiated and named “Three Discipline Collaborative Radiation Therapy (3DCRT).” The idea for this format originated from an NIH‐funded educational course called “Integration of Biology and Physics into Radiation Oncology (IBPRO)” which fostered multidisciplinary clinical and research collaboration. This course successfully generated multidisciplinary collaborative relationships and associated research productivity, and one of its hallmark educational activities was the multidisciplinary group debate.^2^ The 3DCRT debate series began by including a radiation oncologist, medical physicist, and radiobiologist on each debate team. The first debate argued the need for more proton treatment facilities in the US,^3^ and subsequent debates argued a variety of controversial topics within radiation oncology. More recently, the 3DCRT debates have broken from the traditional participants and have begun involving others, such as patient advocates or clinical trainees within these three person teams. However, we continue to require the same collaborative approach, specifically that the debate product not be a set of standalone arguments from each discipline, but rather a true “team‐science” format in which each team produces coherent arguments which knit together the expertise afforded by each individual discipline. To date, thirteen 3DCRT articles have been published with several more in process, and we are currently in the process of initiating team debates outside of the discipline of radiation oncology. We hope this format is not only engaging to the readership but fosters further collaborative science and clinical practice.

Many more interesting and controversial Parallel Opposed and 3DCRT debates are listed in the Virtual Issue of “Debates in Medical Physics” on the JACMP website (Debates in Medical Physics: Journal of Applied Clinical Medical Physics (wiley.com)). Moreover, this debate series has inspired a live “JACMP debate” session at the 2023 and 2024 annual AAPM meetings, and we hope that its tremendous initial success will make it a recurring session at future meetings.

Debates have been commonly adopted by educators as an effective active learning technique. Engaging in these debates exercises critical thinking, a fundamental skill necessary for medical physics practice. Experts present diverse perspectives on a given topic, providing their audience with an opportunity to both utilize their scientific acumen and apply the art of balancing conflicting objectives. Celebrating this milestone of our debate series, which has now been running for almost 10 years, provides us with an opportunity to reflect on the contributions of those authors who have selflessly inspired the field of medical physics and radiation oncology with their wit and wisdom. Their ideas and arguments not only help us exercise our own critical thinking, but also foster further contemplation, collaboration, and innovation within our profession.

## CONFLICT OF INTEREST STATEMENT

The authors declare no conflicts of interest.
